# Theoretical Study of Lumped Parameter Circuits and Multiconductor Transmission Lines for Time-Domain Analysis of Electromagnetic Noise

**DOI:** 10.1038/s41598-018-36383-3

**Published:** 2019-01-15

**Authors:** Masayuki Abe, Hiroshi Toki

**Affiliations:** Graduate School of Engineering Science, Osaka University, Toyonaka, Osaka 560-8531 Japan

## Abstract

In order to find the origins of electromagnetic noise in the time domain, we formulate a system of lumped parameter circuits and multiconductor transmission lines (MTL). We present a discretized approach to treat any lumped parameter circuits and MTL systems, and the boundary conditions between these systems, where the lumped parameter circuits are described by coupled differential equations, and the MTL systems by coupled partial-differential equations. The introduction of the time-domain impedance and the element matrices enables us to perform a time-domain analysis that includes dependent sources and the coupling devices in the framework of the circuit theory. For three-line systems, we are able to calculate the coupling of the normal, common, and antenna modes, and to find out methods to reduce the noise.

## Introduction

Electromagnetic noise is troublesome; it not only affects the transmission of signals, but also causes a malfunction. An integrated circuit (IC) has electromagnetic noise that interferes with the signal, and an important area of research for IC is electromagnetic compatibility (EMC)^1^. Many scientists have studied this phenomenon by taking a symptomatic approach to systems from the IC level to power lines^2–6^. In the attempt to locate the sources of electromagnetic noise, many authors have considered environments in terms of conductors in the ground and power plane and/or the signal lines. The electromagnetic noise was studied also experimentally using printed circuit boards (PCB) and the results were analyzed by introducing a phenomenological model^7,8^. In principle, we should be able to calculate the origin of the electromagnetic noise using the multi-conductor transmission line (MTL) theory based on the Heaviside transport equations^9^. There are many studies in this line of thoughts in various research fields as the performance of multi-wire systems and motor-stater winding-coils with high frequency signals^10,11^. However, it is not easy to identify the sources of the noise in the manipulation of the MTL equations.

In the field of accelerator physics, one successful attempt was made for the reduction of electromagnetic noise in a power supply for a heavy ion medical particle accelerator in Chiba (HIMAC)^12^. The key concept for the noise reduction was to symmetrically configure the three-conductor transmission lines consisting of two circuit lines and one ground line. In addition, the circuit elements should be symmetrically arranged around the ground line in order to decouple the normal and common modes. This method reduced the noise to the level of *N*/*S* = 10^−6^ ^12^. The geometrical symmetry of the multiconductor transmission-lines (MTL) and that of the lumped parameter circuit elements were both found to be critical for the success of the reduction of noise^13^.

In order to understand the role of the symmetrization for the reduction of noise, there was an attempt to describe theoretically the three-conductor transmission lines^14^. In an effort to introduce the variables in the normal and common modes in the MTL equations, it was realized that the use of the concept of capacitor made the manipulation of the MTL equations very difficult and unclear. Instead, if we rewrite the MTL equations in terms of the coefficients of potential, we were able to express the MTL equations straightforwardly and found the condition to decouple the normal mode from the common mode^14^. The decoupling condition states that the two main circuit lines have to be symmetrically arranged around the third ground line, and all the electric components have to be arranged symmetrically around the third line as found in the accelerator science.

For the purpose of simulating the mechanism of noise generation and its reduction, we have to facilitate these findings by introducing lumped parameter circuits connected with the MTL system. In this formalism, we have to make the concept of the common mode clear, and to define the noise, which is not included in the standard MTL theory proposed by Heaviside a century ago. To this end, we should derive the MTL equations from the Maxwell equations, and identify the noise terms by deriving the Heaviside telegraphic equations by several approximations. Those terms which are neglected in the process of reducing the full equations down to the Heaviside equations are to be identified as noise terms.

**Figure 1:**
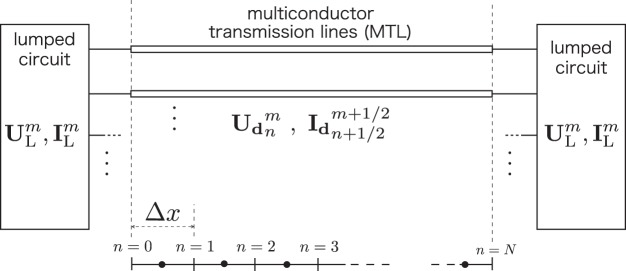
A coupled system of lumped parameter circuits and multi-conductor transmission lines. Model of discretization at the boundary of lumped parameter circuit with any number of MTLs, where lumped parameter circuits are connected at both ends. The finite-difference time-domain (FDTD) method is used to solve for the transmission of signals and emission of noise in the MTLs. Lumped circuits consist of passive elements and independent and dependent sources and coupled elements. Here, **U** and **I** are the potential and current vectors and the subscript *d* represents the MTL, and *m* and *n* denote discretized time and space, respectively.

We have to derive boundary equations for both the MTL equations and the lumped parameter circuits, which are shown in Fig. 1. The boundary conditions play an important role for two kinds of differential equations to be solved simultaneously: ordinary coupled differential equations for the lumped parameter circuit and partial differential equations for the MTL system^15^. Recently, we have introduced the fundamental concept for the boundary conditions of lumped parameter circuits and the MTL system^16,17^. There were, however, several ingredients, whose details were not described explicitly. One is the coupling devices such as dependent power sources in the lumped parameter circuit and another is the explicit algorithm for the treatment of the radiation terms.

A formalism similar to the present method is the partial element equivalent circuits (PEEC) method^18,19^, which uses the retarded potentials with the Green’s functions in the Lorenz gauge of Maxwell’s equations and introduces the concept of small cells for conductors. In the PEEC method, in order to perform a SPICE-like simulation, the distributed conductive materials are divided into small cells, and each cell is regarded as a set of connected lumped elements of inductance and capacitor^18,20,21^. This method is, however, not straightforward to express the common and normal modes that need to be considered when evaluating electromagnetic noise. Hence, in the PEEC method, it is difficult to find the ideal configurations for the reduction of electromagnetic noise. In addition, the concept of the PEEC method is far from the standard Heaviside telegraphic equation, which is commonly used for the MTL system^9^. On the other hand, in our method, the use of the characteristic impedance and the incidence matrix with the standard circuit theory enables us to connect to the telegraphic equation and to evaluate the coupling of the normal and common modes explicitly^16^.

The aim of this paper is to use a circuit theory to obtain discretized equations for the boundary conditions between a lumped parameter circuit and MTL system in order to perform a time-domain analysis of electromagnetic noise. In the first part of Section 2, we introduce the incidence-potential equation (IPE) for the lumped parameter circuit for node potentials and element currents. The IPE includes an incidence and an impedance matrices, which are sufficient to describe a lumped parameter circuit. We then add controlled sources and coupling devices to the IPE. In Section 3, we discretize the matrix equation so that it can be used for a time-domain analysis of the lumped parameter circuit, which includes capacitors and inductors. In Section 4, the matrix equations are combined with the discretized equations for the MTL system. This provides a boundary condition for the connection of the lumped parameter circuit to the MTL system.

## Matrix Equation for Lumped Circuit Including Controlled Sources And Coupling Devices

We shall begin with construction of coupled equations for the lumped parameter circuit in order to connect smoothly with the MTL equations at the boundary. The potential and current are the independent variables in the MTL equations^9,15^, and it is better to use node potentials rather than element voltages in the lumped parameter circuit. For the lumped parameter circuit, the matrix equation at the boundary must satisfy Kirchhoff’s current and voltage laws (KCL and KVL, respectively), and the branch constitutive equation (BCE).

We consider first a lumped parameter circuit with independent power sources without the coupled elements as dependent power sources. We introduce the incidence matrix **A**_*T*_, where the column numbers describe element currents associated with each node point numbered as a row number in the circuit. **A**_*T*_ can be separated into submatrices: **A**_*T*_ = (**A A**_*J*_), where the column of **A** represents the circuit elements (resistors, capacitances, inductances, and voltage supplies), and that of **A**_*J*_ the independent current sources. The element current vector **I**_0_ can then be separated into two subvectors: circuit element currents **I** and source currents **J**; **I**_*T*_ = (**I J**)^*T*^. The current conservation relation (KCL) provides **A**_*T*_**I**_*T*_ = **0**, which provides the following equation for the element currents,

1 $${\bf{A}}{\bf{I}}{\boldsymbol{+}}{{\bf{A}}}_{{\bf{J}}}{\bf{J}}={\bf{0}}.$$

We note that the transpose of the incidence matrix **A** expresses the relation between the node potential vector **U** and the element voltages **V**; **A**^*T*^**U** = **V**, which eventually provides the KVL equation. The branch constitutive equation (BCE) is written as **V** = **ZI** + **E**, where the passive elements are expressed by the impedance matrix **Z**, including the independence voltage sources expressed by the voltage source vector **E**. We thus have the relation,

2 $${{\bf{A}}}^{T}{\bf{U}}-{\bf{ZI}}={\bf{E}}.$$

The sparse-tableau type form^22^ is obtained from these formulae by bringing the unknown quantities **U** and **I** in the left hand side, while the current sources **J** and the voltage sources **E** are known and brought to the right hand side:

3 $$(\begin{array}{cc}{{\bf{A}}}^{T} & -{\bf{Z}}\\ {\bf{0}} & {\bf{A}}\end{array})(\begin{array}{c}{\bf{U}}\\ {\bf{I}}\end{array})=(\begin{array}{c}{\bf{E}}\\ -{{\bf{A}}}_{J}{\bf{J}}\end{array}).$$

This is the fundamental equation to be used for the boundary equation between the lumped parameter circuit and the MTL system, and we name this Eq. (3) as Incidence-Potential Equation (IPE).

**Figure 2:**
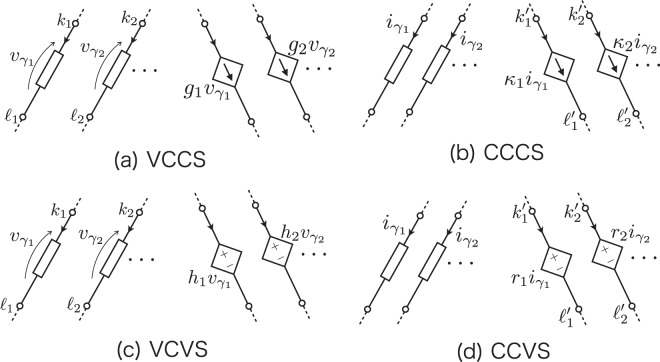
Connections between controlling and controlled elements: (**a**) voltage-controlled current source (VCCS), (**b**) current-controlled current source (CCCS), (**c**) voltage-controlled voltage source (VCVS), and (**d**) current-controlled current source (CCVS). The element numbers and the node potentials are indicated.

We formulate next the dependent power supplies as shown in Fig. 2 to complete the fundamental structure of IPE. The treatment of the dependent power sources can be applied to other coupling devices such as mutual inductances and the transistors as the non-linear devices. We begin with the dependent current sources, where the incidence matrix **A**_*T*_ would be rewritten as the one that includes submatrices **A**_VCCS_ for the VCCS and **A**_CCCS_ for the CCCS. Therefore, **A**_*T*_ has four submatrices: **A**_*T*_ = (**A A**_*J*_**A**_VCCS_**A**_CCCS_). We can write the current vector as **I**_*T*_ = (**I J J**_VCCS_**J**_CCCS_)^*T*^, where **J**_VCCS_ and **J**_CCCS_ are the VCCS and CCCS vectors, respectively. The KCL equation is written as,

4 $${\bf{AI}}+{{\bf{A}}}_{J}{\bf{J}}+{{\bf{A}}}_{{\rm{VCCS}}}{{\bf{J}}}_{{\rm{VCCS}}}+{{\bf{A}}}_{{\rm{CCCS}}}{{\bf{J}}}_{{\rm{CCCS}}}=0.$$

Since the third and fourth terms of Eq. (4) are controlled sources, they can be expressed by the reference voltages and currents as the controlling elements.

As shown in Fig. 2(a), the current values of the VCCS refer to the respective element voltages ${\upsilon }_{{\gamma }_{1}},{\upsilon }_{{\gamma }_{2}},\cdots$, and thus they can be expressed using the node potentials as ${g}_{1}{v}_{{\gamma }_{1}}={g}_{1}({u}_{{k}_{1}}-{u}_{{\ell }_{1}}),{g}_{2}{v}_{{\gamma }_{2}}={g}_{2}({u}_{{k}_{2}}-{u}_{{\ell }_{2}}),\cdots$. Here ${g}_{{\gamma }_{1}},{g}_{{\gamma }_{2}},\cdots$ are transconductances of the VCCS. This allows us to write the following equation using the potential vector **U**:

5 $$\begin{array}{ccc}{{\bf{A}}}_{{\rm{V}}{\rm{C}}{\rm{C}}{\rm{S}}}{{\bf{J}}}_{{\rm{V}}{\rm{C}}{\rm{C}}{\rm{S}}} & = & {{\bf{A}}}_{{\rm{V}}{\rm{C}}{\rm{C}}{\rm{S}}}(\begin{array}{c}{g}_{1}({u}_{{k}_{1}}-{u}_{{\ell }_{1}})\\ {g}_{2}({u}_{{k}_{2}}-{u}_{{\ell }_{2}})\\ \vdots \end{array})\\  & = & {{\bf{A}}}_{{\rm{V}}{\rm{C}}{\rm{C}}{\rm{S}}}\,{\bf{G}}{{\bf{A}}{}^{{\boldsymbol{^{\prime} }}}}_{{\rm{V}}{\rm{C}}{\rm{C}}{\rm{S}}}^{T}\,{\bf{U}}={{\bf{D}}}_{{\rm{V}}{\rm{C}}{\rm{C}}{\rm{S}}}\,{\bf{U}}.\end{array}$$

here, **A**_VCCS_ expresses the place of the dependent current sources in the circuit, **G** is a diagonal matrix in which the elements are *g*_1_, *g*_2_, …. The sub-incidence matrix ${{\bf{A}}{\boldsymbol{^{\prime} }}}_{{\rm{VCCS}}}^{T}$ relates the dependent current sources to the controlling voltages expressed by the corresponding potentials **U**. The number of row of ${{\bf{A}}{\boldsymbol{^{\prime} }}}_{{\rm{VCCS}}}^{T}$ is the number of the dependent current sources. ${{\bf{A}}}_{{\rm{VCCS}}}{\bf{G}}{{\bf{A}}{\boldsymbol{^{\prime} }}}_{{\rm{VCCS}}}^{T}$ is written by a matrix **D**_VCCS_, which is referred to dependent source matrix of VCCS.

We obtain a similar expression for the fourth term **A**_CCCS_**J**_CCCS_. The CCCSs (Fig. 2(b)) refer to the respective controlling current ${i}_{{\gamma }_{j}}$ with coupling constants *κ*_1_,*κ*_2_, …. We can then write the CCCS term as

6 $$\begin{array}{ccc}{{\bf{A}}}_{{\rm{C}}{\rm{C}}{\rm{C}}{\rm{S}}}{{\bf{J}}}_{{\rm{C}}{\rm{C}}{\rm{C}}{\rm{S}}} & = & {{\bf{A}}}_{{\rm{C}}{\rm{C}}{\rm{C}}{\rm{S}}}(\begin{array}{c}{\kappa }_{1}{i}_{{\gamma }_{1}}\\ {\kappa }_{2}{i}_{{\gamma }_{2}}\\ \vdots \end{array})\\  & = & {{\bf{A}}}_{{\rm{C}}{\rm{C}}{\rm{C}}{\rm{S}}}\,{\bf{K}}{{\bf{B}}{}^{{\boldsymbol{^{\prime} }}}}_{{\rm{C}}{\rm{C}}{\rm{C}}{\rm{S}}}^{T}\,{\bf{I}}={{\bf{D}}}_{{\rm{C}}{\rm{C}}{\rm{C}}{\rm{S}}}\,{\bf{I}}.\end{array}$$

here, **A**_CCCS_ expresses the place of the dependent current sources in the circuit, and **K** is a diagonal matrix consisting of the coupling constants *κ*_1_,*κ*_2_, …. The sub-incidence matrix ${{\bf{B}}{\boldsymbol{^{\prime} }}}_{{\rm{CCCS}}}^{T}$ relates the dependent current sources to the controlling currents expressed by the corresponding currents **I**. ${{\bf{A}}}_{{\rm{CCCS}}}{\bf{K}}{{\bf{B}}{\boldsymbol{^{\prime} }}}_{{\rm{CCCS}}}^{T}$ is written by a matrix **D**_CCCS_, which is referred to dependent source matrix for CCCS.

In order to derive the dependent source matrices for the VCVS (Fig. 2(c)) and the CCVS (Fig. 2(d)), we rewrite **E** as **E** + **E**_VCVS_ + **E**_CCVS_, where **E**_VCVS_ and **E**_CCVS_ are the voltage vectors for VCVS and CCVS, respectively. The rewritten **E** expresses independent voltage sources. By combining the KVL and BCE, we obtain the following equation.

7 $${{\bf{A}}}^{T}{\bf{U}}-{\bf{ZI}}={\bf{E}}+{{\bf{E}}}_{{\rm{VCVS}}}+{{\bf{E}}}_{{\rm{CCVS}}}.$$

Both **E**_VCVS_ and **E**_CCVS_ can be rewritten by using the potential **U** and current **I** vectors.

8 $$\begin{array}{ccc}{{\bf{E}}}_{{\rm{V}}{\rm{C}}{\rm{V}}{\rm{S}}} & = & (\begin{array}{c}0\\ \vdots \\ {h}_{1}{\upsilon }_{{\gamma }_{1}}\\ \vdots \\ {h}_{2}{\upsilon }_{{\gamma }_{2}}\\ \vdots \end{array})=(\begin{array}{c}0\\ \vdots \\ {h}_{1}({u}_{{k}_{1}}-{u}_{{\ell }_{1}})\\ \vdots \\ {h}_{2}({u}_{{k}_{2}}-{u}_{{\ell }_{2}})\\ \vdots \end{array})\\  & = & {{\bf{B}}}_{{\rm{V}}{\rm{C}}{\rm{V}}{\rm{S}}}\,{\bf{H}}{{\bf{A}}{}^{{\boldsymbol{^{\prime} }}}}_{{\rm{V}}{\rm{C}}{\rm{V}}{\rm{S}}}^{T}\,{\bf{U}}={{\bf{D}}}_{{\rm{V}}{\rm{C}}{\rm{V}}{\rm{S}}}\,{\bf{U}}.\end{array}$$

We can write the CCVS case similarly as:

9 $${{\bf{E}}}_{{\rm{C}}{\rm{C}}{\rm{V}}{\rm{S}}}=(\begin{array}{c}0\\ \vdots \\ {r}_{1}{i}_{{\gamma }_{1}}\\ \vdots \\ {r}_{2}{i}_{{\gamma }_{2}}\\ \vdots \end{array})={{\bf{B}}}_{{\rm{C}}{\rm{C}}{\rm{V}}{\rm{S}}}\,{\bf{R}}{{\bf{B}}{}^{{\boldsymbol{^{\prime} }}}}_{{\rm{C}}{\rm{C}}{\rm{V}}{\rm{S}}}^{T}\,{\bf{I}}={{\bf{D}}}_{{\rm{C}}{\rm{C}}{\rm{V}}{\rm{S}}}\,{\bf{I}}.$$

Here, **H** and **R** are diagonal matrices that include the voltage transfer ratios and transresistances, respectively. The other sub-matrices, **B**_VCVS_, **A**_VCVS′_, **B**_CCVS_, and **B**_CCVS′_, can be built in the same manner as described above. **D**_VCVS_ and **D**_CCVS_ are the dependent source matrices of VCVS and CCVS, respectively.

We can now use all the above expressions containing **U** and **I** to rewrite the IPE (3) to include the controlled sources.

10 $$\{(\begin{array}{cc}-{{\bf{D}}}_{{\rm{V}}{\rm{C}}{\rm{V}}{\rm{S}}} & -{{\bf{D}}}_{{\rm{C}}{\rm{C}}{\rm{V}}{\rm{S}}}\\ {{\bf{D}}}_{{\rm{V}}{\rm{C}}{\rm{C}}{\rm{S}}} & {{\bf{D}}}_{{\rm{C}}{\rm{C}}{\rm{C}}{\rm{S}}}\end{array})+(\begin{array}{cc}{{\bf{A}}}^{T} & -{\bf{Z}}\\ {\bf{0}} & {\bf{A}}\end{array})\}(\begin{array}{c}{\bf{U}}\\ {\bf{I}}\end{array})=(\begin{array}{c}{\bf{E}}\\ -{{\bf{A}}}_{J}{\bf{J}}\end{array}).$$

We base on this Eq. (10) for further discussion to express the boundary condition between lumped parameter circuits with a MTL system. We note here that this Eq. (10) can also be used to solve for the steady state AC and DC of a lumped parameter circuit. In the AC steady state, *R*, 1/(*jωC*), and *jωL* can be used as elements in the **Z** matrix. In this case, by calculating the inverse of the matrix on the right-hand side of Eq. (10), we obtain the node potentials **U** and the element currents **I**. A mutual inductor *M* can be regarded as two CCVSs that have a combined transresistance of *jωM* in the AC steady state.

## Discretized IPE for time-domain circuit analysis

We want to write the IPE (10) in the time domain for the analysis of noise. To this end, we rewrite first the BCE using the second-order Adams-Moulton formula^23^:

11 $$\begin{array}{cc}R: & {V}^{m+1}-R{I}^{m+1}=-\,({V}^{m}-R{I}^{m}),\\ C: & {V}^{m+1}-\frac{{\rm{\Delta }}t}{2C}{I}^{m+1}=-\,((\,-\,1){V}^{m}-\frac{{\rm{\Delta }}t}{2C}{I}^{m}),\\ L: & {V}^{m+1}-\frac{2L}{{\rm{\Delta }}t}{I}^{m+1}=-\,({V}^{m}-\,(\,-\,1)\frac{2L}{{\rm{\Delta }}t}{I}^{m}),\\ E: & {V}^{m+1}=-\,{V}^{m}+({E}^{m+1}+{E}^{m}).\end{array}$$

Here, *m* is the discretized time, and the above equations provide voltages and currents at an advanced time *m* + 1 in terms of the present time quantities for each element. For the time-domain analysis, the time-domain impedances can be defined as elements of the impedance matrix **Z**^16^. From Eq. (11), the time-domain impedances are as follows: for the resistor, it is *R*; for the capacitor, Δ*t*/(2*C*); for the inductor, 2*L*/(Δ*t*); and for the independent voltage source, 0. The factors of (−1) in Eq. (11) come from the differential relation of the capacitor and inductor. Using the above expressions (11), we obtain a discretize IPE from Eq. (10) as follows:

12 $$\begin{array}{c}\{(\begin{array}{cc}-{{\bf{D}}}_{{\rm{V}}{\rm{C}}{\rm{V}}{\rm{S}}} & -{{\bf{D}}}_{{\rm{C}}{\rm{C}}{\rm{V}}{\rm{S}}}\\ {{\bf{D}}}_{{\rm{V}}{\rm{C}}{\rm{C}}{\rm{S}}} & {{\bf{D}}}_{{\rm{C}}{\rm{C}}{\rm{C}}{\rm{S}}}\end{array})+(\begin{array}{cc}{{\bf{A}}}^{T} & -{\bf{Z}}\\ {\bf{0}} & {\bf{A}}\end{array})\}(\begin{array}{c}{{\bf{U}}}^{m+1}\\ {{\bf{I}}}^{m+1}\end{array})\\ \begin{array}{ccc} & = & \{(\begin{array}{cc}{{\bf{D}}}_{{\rm{V}}{\rm{C}}{\rm{V}}{\rm{S}}} & {{\bf{D}}}_{{\rm{C}}{\rm{C}}{\rm{V}}{\rm{S}}}\\ {\bf{0}} & {\bf{0}}\end{array})-(\begin{array}{cc}\varepsilon {{\bf{A}}}^{T} & -{\boldsymbol{\delta }}{\bf{Z}}\\ {\bf{0}} & {\bf{0}}\end{array})\}(\begin{array}{c}{{\bf{U}}}^{m}\\ {{\bf{I}}}^{m}\end{array})\\  &  & +(\begin{array}{c}{{\bf{E}}}^{m+1}+{{\bf{E}}}^{m}\\ -{{\bf{A}}}_{J}{{\bf{J}}}^{m+1}\end{array}).\end{array}\end{array}$$

Here, ***ε*** and ***δ*** are diagonal matrices. The *γ* th element *ε*_*γγ*_ of the matrix ***ε*** depends on the circuit elements. That is,

13 $${\varepsilon }_{\gamma \gamma }=\{\begin{array}{cc}-\,1 & (\text{for}\,\text{capacitors})\\ 1 & (\text{for}\,{\rm{o}}{\rm{t}}{\rm{h}}{\rm{e}}{\rm{r}}\,\text{elements})\end{array}.$$

The elements of matrix ***δ*** are

14 $${\delta }_{\gamma \gamma }=\{\begin{array}{cc}-\,\,1 & (\text{for}\,\text{inductors})\\ 1 & (\text{for}\,{\rm{o}}{\rm{t}}{\rm{h}}{\rm{e}}{\rm{r}}\,\text{elements})\end{array}.$$

Since the origin of the dependent voltage sources **D**_CCVS_ and **D**_VCVS_ are the same as the independent voltage sources **E**, we ought to introduce the minus sign in the right hand side of the above equation. As for the dependent current sources **D**_CCCS_ and **D**_VCCS_, the corresponding terms in the right hand side are set zero as the case of the independent current sources **J**. The sign changes in Eq. (11) are considered by ***ε*** for capacitors and ***δ*** for inductors. Since this Eq. (12) is obtained for the inclusion of time domain impedance as capacitors and inductors, we name this as Time-Domain IPE.

We note here that SPICE treats the quantities in the right hand sides in Eq. (11) as external sources for each element. In our approach, we prefer to treat these quantities explicitly as the consequence of the discretization of the time dependent equations as shown in the time domain IPE (12). This is because MTL equations of distributed circuit are given in the coupled differential equations for potentials and currents in the standard FDTD method^9^, and the expression in time dependent IPE (12) can naturally express the boundary condition between the lumped parameter circuit and the MTL system as discussed in the next section.

## MTL equations with retardation effect

We write here the MTL equations including the retardation effect^15,16^. We treat first the retarded scalar potential $U(\vec{r},\,t)$ in the conductor, which is expressed by the charge density $q(\vec{r},\,t)$ in the conductor^16^.

15 $$U(\vec{r},t)=\frac{1}{4\pi \varepsilon }\int {d}^{3}r^{\prime} \frac{q({\vec{r}}^{{\rm{^{\prime} }}}t^{\prime} )}{|\vec{r}-{\vec{r}}^{^{\prime} }|},$$

where $t^{\prime} =t-|\vec{r}-{\vec{r}}^{^{\prime} }|/c$ represents the effect of the retardation, and *c* is the velocity of the electromagnetic wave. We consider the case of a MTL system and introduce *n* thin wires. These thin wires have radii *a*_*i*_ and arranged with the mutual distances *a*_*ij*_ between wires with *i* = 1, .. *n* as shown in Fig. 1. We introduce the charge per length by integrating the charge density *q* by the cross section assuming that the charge density is unchanged within the cross section of area *S*:

16 $${Q}_{i}(x^{\prime} ,t^{\prime} )={\int }_{S}dy^{\prime} dz^{\prime} {q}_{i}(x^{\prime} ,y^{\prime} ,z^{\prime} ,t^{\prime} ).$$

We introduce a mean distance *d* using the following relation:

17 $$\frac{1}{\sqrt{{(x-x^{\prime} )}^{2}+{d}^{2}}}=\frac{1}{S}{\int }_{S}dy^{\prime} dz^{\prime} \frac{1}{\sqrt{{(x-x^{\prime} )}^{2}+{y^{\prime} }^{2}+{(a-z^{\prime} )}^{2}}}.$$

With these definitions, we can write the potential at a surface (*y* = 0, *z* = *a*) of a wire as

18 $${U}_{i}(x,0,a,t)=\frac{1}{4\pi \varepsilon }\sum _{j=1}^{n}\int dx{\rm{^{\prime} }}\frac{{Q}_{j}(x{\rm{^{\prime} }},t-|x-x{\rm{^{\prime} }}|/c)}{\sqrt{{(x-x{\rm{^{\prime} }})}^{2}+{d}_{ij}^{2}}}.$$

Here, the mean distances *d*_*ij*_ are close to the radius of the *i*-th wire *a*_*i*_ for *i* = *j*, and close to the distance *a*_*ij*_ between the *i*-th and *j*-th wires. We have the similar expression for the vector potential $\vec{A}$ in terms of the current per length *I*. Using the same procedure we arrive at the expression:

19 $${A}_{i}(x,0,a,t)=\frac{\mu }{4\pi }\sum _{j=1}^{n}\int d{x}^{{\rm{^{\prime} }}}\frac{{I}_{j}({x}^{{\rm{^{\prime} }}},t-|x-{x}^{{\rm{^{\prime} }}}|/c)}{\sqrt{{(x-{x}^{{\rm{^{\prime} }}})}^{2}+{d}_{ij}^{2}}}.$$

Here, we consider the case that the wire is thin and the direction of the current and the vector potential is the *x*-direction and drop the suffix *x* in the above expression.

We need two more relations for each wire in order to complete the MTL equation. One is the continuity equation for the relation between the current and the charge:

20 $$\frac{\partial {Q}_{i}(x,t)}{\partial t}+\frac{\partial {I}_{i}(x,t)}{\partial x}=0.$$

Another relation is provided by the Ohm’s law for the electric field *E*_*x*_ and the current *I*_*x*_, *E*_*x*_ = *RI*_*x*_ for each wire. We drop the *x* suffix and express the electric field in terms of the scalar and vector potentials:

21 $$-\frac{\partial {U}_{i}(x,t)}{\partial x}-\frac{\partial {A}_{i}(x,t)}{\partial t}={R}_{i}{I}_{i}(x,t).$$

Here, *R*_*i*_ is the resistance per length for the *i*-th wire.

We can express the four relations in terms of two relations by taking the partial derivative of the potentials with respect to time. We get one relation for the scalar potential by using the continuity Eq. (20):

22 $$\frac{{\rm{\partial }}{U}_{i}(x,t)}{{\rm{\partial }}t}=-\,\frac{1}{4\pi \varepsilon }\sum _{j=1}^{n}\int dx^{\prime} \frac{1}{\sqrt{{(x-x^{\prime} )}^{2}+{d}_{ij}^{2}}}\frac{{\rm{\partial }}{I}_{j}(x^{\prime} ,t-|x-x^{\prime} |/c)}{{\rm{\partial }}x^{\prime} }.$$

Here, we have dropped the *y*, *z* coordinates in the scalar potential and write only the *x* coordinate. As for the second equation, we obtain first the time derivative of the vector potential, and use the Ohm’s law relation (21) to arrive at the following relation:

23 $$\frac{{\rm{\partial }}{U}_{i}(x,t)}{{\rm{\partial }}x}=-\,\frac{\mu }{4\pi }\sum _{j=1}^{n}\int dx^{\prime} \frac{1}{\sqrt{{(x-x^{\prime} )}^{2}+{d}_{ij}^{2}}}\frac{{\rm{\partial }}{I}_{j}(x^{\prime} ,t-|x-x^{\prime} |/c)}{{\rm{\partial }}t}-{R}_{i}{I}_{i}(x,t).$$

We note here that the case of *R*_*i*_ = 0 corresponds to the lossless line equation.

We introduce the finite-difference time-domain (FDTD) method to solve the MTL equations numerically, where we introduce the integer numbers *x* = *k*Δ*x* for *x* coordinate and *t* = *m*Δ*t* for time:

24 $$\frac{{U}_{i,k}^{m+1}-{U}_{i,k}^{m}}{{\rm{\Delta }}t}=-\,\frac{1}{4\pi \varepsilon }\sum _{j,k^{\prime} }\frac{{\rm{\Delta }}x}{\sqrt{{((k-k^{\prime} ){\rm{\Delta }}x)}^{2}+{d}_{ij}^{2}}}\frac{1}{{\rm{\Delta }}x}({I}_{j,k^{\prime} +1/2}^{m+1/2-|k-k^{\prime} |\alpha }-{I}_{j,k^{\prime} -1/2}^{m+1/2-|k-k^{\prime} |\alpha }).$$

Here, we write *α* = Δ*x*/*c*Δ*t*, where *α* is related with the stability of the FDTD method and for the one-dimensional case *α* ≥ 1. In the above expression, we have neglected the effect of the size of the wires and the distances between wires in the retardation time. When these sizes and distances become large, we ought to formulate the MTL system as a two (three) dimensional problem. The retardation time is calculated at the place of the charge. In the above expression, there appears the terms *I*_*j*,−1/2_ and *I*_*j*, *N* + 1/2_, which are outside of the wire. In this case we define them as

25 $$\begin{array}{c}{I}_{j,-1/2}^{m+1/2-k\alpha }\to {I}_{j,0}^{m+1/2-k\alpha }=\frac{1}{2}({I}_{j,0}^{m+1-k\alpha }+{I}_{j,0}^{m-k\alpha }),\\ {I}_{j,N+1/2}^{m+1/2-|k-N|\alpha }\to {I}_{j,N}^{m+1/2-|k-N|\alpha }=\frac{1}{2}({I}_{j,N}^{m+1/2-|k-N|\alpha }+{I}_{j,N}^{m-|k-N|\alpha }).\end{array}$$

With this replacement, we ought to take the distance Δ*x* → Δ*x*/2. This replacement is compensated by the 1/2 for the weight of the edge coordinate in the trapezoidal integral formula.

We can write the other equation in the similar manner.

26 $$\begin{array}{ccc}\frac{{U}_{i,k+1}^{m}-{U}_{i,k}^{m}}{{\rm{\Delta }}x} & = & -\frac{\mu }{4\pi }\sum _{j,k^{\prime} }\frac{{\rm{\Delta }}x}{\sqrt{{((k-k^{\prime} ){\rm{\Delta }}x)}^{2}+{d}_{ij}^{2}}}\frac{1}{{\rm{\Delta }}t}({I}_{j,k^{\prime} +1/2}^{m+1/2-|k-k^{\prime} |\alpha }-{I}_{j,k^{\prime} +1/2}^{m-1/2-|k-k^{\prime} |\alpha })-\,\,\frac{1}{2}{R}_{i}({I}_{i,k+1/2}^{m+1/2}+{I}_{i,k+1/2}^{m-1/2}).\end{array}$$

We introduce local coefficients of potential *P*_*ik*, *jk*′_ and local coefficients of inductance *L*_*ik*, *jk*′_ as

27 $${P}_{ik,jk^{\prime} }=\frac{1}{4\pi \varepsilon }\frac{{\rm{\Delta }}x}{\sqrt{{((k-k^{\prime} ){\rm{\Delta }}x)}^{2}+{d}_{ij}^{2}}},$$

28 $${L}_{ik,jk^{\prime} }=\frac{\mu }{4\pi }\frac{{\rm{\Delta }}x}{{((k-k^{\prime} ){\rm{\Delta }}x)}^{2}+{d}_{ij}^{2}}.$$

We have to pay a special attention on this expression for *k* = *k*′, since these coefficients become very large. We should perform the integration within the small distance Δ*x* rigorously:

29 $${P}_{i,j}=\frac{1}{4\pi \varepsilon }{\int }_{-{\rm{\Delta }}x/2}^{{\rm{\Delta }}x/2}dx^{\prime} \frac{1}{\sqrt{{x^{\prime} }^{2}+{d}_{ij}^{2}}}.$$

We have a similar expression for *L*_*i*, *j*_. We note here that these local coefficients of potential and inductance are to be contrasted from the global coefficients of potential *P*, which are inverse of coefficients of capacitance *C* and the global coefficients of inductance *L* of the Heaviside telegraphic equations. These global coefficients are obtained by integrating over the full length of the wires, and use only the currents at the position of the potentials in the telegraphic equations.

We write the above equations in a compact form by introducing the potential vector (**U**_d_) and current vector (**I**_d_) of the MTL in the FDTD method for the MTL system^16^. Here, the bold potential and current denote the quantities of all the transmission lines: **U**_d_ = (U_d1_, U_d2_,...)^*T*^ and **I**_d_ = (I_d1_, I_d2_,...)^*T*^, representing all the transmission lines.

30 $$\frac{{{\bf{U}}}_{{{\rm{d}}}_{k}}^{m+1}-{{\bf{U}}}_{{{\rm{d}}}_{k}}^{m}}{{\rm{\Delta }}t}=-{\bf{P}}\frac{{{\bf{I}}}_{{{\rm{d}}}_{k+1/2}}^{m+1/2}-{{\bf{I}}}_{{{\rm{d}}}_{k-1/2}}^{m+1/2}}{{\rm{\Delta }}x}+\frac{{\mathop{{\bf{U}}}\limits^{ \sim }}_{k}^{m+1}-{\mathop{{\bf{U}}}\limits^{ \sim }}_{k}^{m}}{{\rm{\Delta }}t},$$

31 $$\frac{{{\bf{U}}}_{{{\rm{d}}}_{k+1}}^{m+1}-{{\bf{U}}}_{{{\rm{d}}}_{k}}^{m+1}}{{\rm{\Delta }}x}=-{\bf{L}}\frac{{{\bf{I}}}_{{{\rm{d}}}_{k+1/2}}^{m+3/2}-{{\bf{I}}}_{{{\rm{d}}}_{k+1/2}}^{m+1/2}}{{\rm{\Delta }}t}-\frac{{\mathop{{\bf{A}}}\limits^{ \sim }}_{k+1/2}^{m+1/2}-{\mathop{{\bf{A}}}\limits^{ \sim }}_{k+1/2}^{m-1/2}}{{\rm{\Delta }}t}-{\bf{R}}\frac{{{\bf{I}}}_{{{\rm{d}}}_{k+1/2}}^{m+3/2}+{{\bf{I}}}_{{{\rm{d}}}_{k+1/2}}^{m+1/2}}{2}.$$

The subscripts *m* (=0, 1, …;) and *k* (=0, 1, …, *N*) indicate the discretized time and position, respectively. The suffix d denotes the potentials and currents of the MTL system. If the finite-difference time-domain (FDTD) method is used, both the position and the time for the potentials are discretized at integer values (${{\bf{U}}}_{{{\rm{d}}}_{n}}^{m}$), while those for the currents are discretized at half-integer values (${{\bf{I}}}_{{{\rm{d}}}_{n+\mathrm{1/2}}}^{m+\mathrm{1/2}}$). **P** = **P**_*kk*_ is the cell potential coefficient matrix, and **L** = **L**_*kk*_ is the cell inductance coefficient matrix, which are determined by the geometry of the transmission lines (29). **R** is a diagonal matrix that indicates the resistance per unit length of the MTL. Both $\mathop{{\bf{U}}}\limits^{ \sim }$ and $\mathop{{\bf{A}}}\limits^{ \sim }$ are nonlocal and retarded terms for the scalar and vector potentials, respectively, of the electromagnetic field around the MTL; these are known as antenna terms^16^.

32 $$\frac{{\mathop{{\bf{U}}}\limits^{ \sim }}_{k}^{m+1}-{\mathop{{\bf{U}}}\limits^{ \sim }}_{k}^{m}}{{\rm{\Delta }}t}=-\sum _{k^{\prime} \ne k}{{\bf{P}}}_{kk^{\prime} }\frac{{{\bf{I}}}_{k^{\prime} +1/2}^{m+1/2-|k-k^{\prime} |\alpha }-{{\bf{I}}}_{k^{\prime} -1/2}^{m+1/2-|k-k^{\prime} |\alpha }}{{\rm{\Delta }}x},$$

33 $$\frac{{\mathop{{\bf{A}}}\limits^{ \sim }}_{k+1/2}^{m+1/2}-{\mathop{{\bf{A}}}\limits^{ \sim }}_{k+1/2}^{m-1/2}}{{\rm{\Delta }}t}=\sum _{k^{\prime} \ne k}{{\bf{L}}}_{kk^{\prime} }\frac{{{\bf{I}}}_{k^{\prime} +1/2}^{m+1/2-|k-k^{\prime} |\alpha }-{{\bf{I}}}_{k^{\prime} +1/2}^{m-1/2-|k-k^{\prime} |\alpha }}{{\rm{\Delta }}t}.$$

The time difference of charge **Q** can be expressed by the spatial difference of the current **I** using the continuity equation. Without the nonlocal terms, the above MTL Eqs (30) and (31) correspond the telegraphic equations, which are the most common form of transmission equations for the MTL system^9^.

The discretized Eqs (12, 30 and 31) can be combined to obtain the boundary condition. The nonlocal and retarded potentials, $\mathop{{\bf{U}}}\limits^{ \sim }$ and $\mathop{{\bf{A}}}\limits^{ \sim }$, can be regarded as the source terms of the MTL equation, since these quantities are written by all the past currents. The upper equation provides the condition at the boundary, and we can write the above equation at the boundary (*k* = 0) as:

34 $${{\bf{U}}}_{{{\rm{d}}}_{0}}^{m+1}-{{\bf{U}}}_{{{\rm{d}}}_{0}}^{m}=-\,\frac{1}{\alpha }{{\bf{Z}}}_{{\rm{d}}}({{\bf{I}}}_{{{\rm{d}}}_{1/2}}^{m+1/2}-\,\frac{1}{2}({{\bf{I}}}_{{{\rm{d}}}_{0}}^{m+1}+{{\bf{I}}}_{{{\rm{d}}}_{0}}^{m}))+({\mathop{{\bf{U}}}\limits^{ \sim }}_{0}^{m+1}-{\mathop{{\bf{U}}}\limits^{ \sim }}_{0}^{m}).$$

Here, the characteristic impedance is **Z**_d_ = **P**/*c*. We can write the similar equation for the right boundary (*n* = *N*).

35 $${{\bf{U}}}_{{{\rm{d}}}_{N}}^{m+1}-{{\bf{U}}}_{{{\rm{d}}}_{N}}^{m}=-\,\frac{1}{\alpha }{{\bf{Z}}}_{{\rm{d}}}(\frac{1}{2}({{\bf{I}}}_{{{\rm{d}}}_{N}}^{m+1}+{{\bf{I}}}_{{{\rm{d}}}_{N}}^{m})-{{\bf{I}}}_{{{\rm{d}}}_{N-1/2}}^{m+1/2})+({\mathop{{\bf{U}}}\limits^{ \sim }}_{N}^{m+1}-{\mathop{{\bf{U}}}\limits^{ \sim }}_{N}^{m}).$$

Using this relation we are able to combine the discretized Eq. (12) with the MTL Eqs (34) and (35) at the boundaries.

For the boundary equations we should generalize the potential vector as:

36 $${{\bf{U}}}^{m+1}=(\begin{array}{c}{{\bf{U}}}_{{{\rm{d}}}_{0/N}}^{m+1}\\ {{\bf{U}}}_{{\rm{L}}}^{m+1}\end{array}).$$

The current vector is:

37 $${{\bf{I}}}^{m+1}=(\begin{array}{c}{{\bf{I}}}_{{\rm{L}}}^{m+1}\\ {{\bf{I}}}_{{{\rm{d}}}_{0/N}}^{m+1}\end{array}).$$

As for the source term, we have to include the retardation term:

38 $${{\bf{E}}}^{m+1}+{{\bf{E}}}^{m}=(\begin{array}{c}{{\bf{E}}}_{{\rm{L}}}^{m+1}+{{\bf{E}}}_{{\rm{L}}}^{m}\\ {\mathop{{\bf{U}}}\limits^{ \sim }}_{{\text{d}}_{0/N}}^{m+1}-{\mathop{{\bf{U}}}\limits^{ \sim }}_{{\text{d}}_{0/N}}^{m}\end{array}).$$

The current source term is written as:

39 $$-{{\bf{A}}}_{{\rm{J}}}{{\bf{J}}}^{m+1}=(\begin{array}{c}-{{\bf{A}}}_{{\rm{J}}}{{\bf{J}}}_{{\rm{L}}}^{m+1}\\ 0\end{array}).$$

Here, the subscript “L” indicates the lumped parameter circuit, which correspond to those without subscripts in Eqs (3, 10 and 12). The suffix 0/*N* denotes the left boundary (*k* = 0) or the right boundary (*k* = *N*). The elements of matrix **Z** are the time-domain impedance of the lumped parameter circuit **Z**_L_ and the characteristic impedances **Z**_d_ of the MTL^9^.

40 $${\bf{Z}}=(\begin{array}{cc}{{\bf{Z}}}_{{\rm{L}}} & {\bf{0}}\\ {\bf{0}} & \frac{1}{\alpha }{{\bf{Z}}}_{{\rm{d}}}\end{array}).$$

Here, the impedance matrix **Z**_d_ for the MTL part has both the diagonal and non-diagonal elements. The current in the right hand side should be defined as

41 $$\begin{array}{ccc}{{\bf{I}}}^{m} & = & (\begin{array}{c}{{\bf{I}}}_{{\rm{L}}}^{m}\\ \frac{1}{2}{{\bf{I}}}_{{{\rm{d}}}_{0/N}}^{m}-{{\bf{I}}}_{{{\rm{d}}}_{\chi }}^{m+1/2}\end{array}),\\ \chi  & = & \{\begin{array}{c}\frac{1}{2}\,\,(\text{for}\,n=0)\,\\ N-\frac{1}{2}\,\,(\text{for}\,n=N)\,\end{array}.\end{array}$$

The diagonal elements of ***ε*** and ***δ*** are different from those in Eqs (13) and (14). Here, they are

42 $${\varepsilon }_{\gamma \gamma }=\{\begin{array}{cc}-1 & (\text{for}\,{\rm{c}}{\rm{a}}{\rm{p}}{\rm{a}}{\rm{c}}{\rm{i}}{\rm{t}}{\rm{o}}{\rm{r}}{\rm{s}}\,{\rm{a}}{\rm{n}}{\rm{d}}\,\text{MTL})\\ 1 & (\text{for}\,{\rm{o}}{\rm{t}}{\rm{h}}{\rm{e}}{\rm{r}}\,\text{elements})\end{array}.$$

43 $${\delta }_{\gamma \gamma }=\{\begin{array}{c}-1\,\,(\text{for}\,\text{inductors})\\ 1\,\,(\text{for}\,{\rm{o}}{\rm{t}}{\rm{h}}{\rm{e}}{\rm{r}}\,{\rm{e}}{\rm{l}}{\rm{e}}{\rm{m}}{\rm{e}}{\rm{n}}{\rm{t}}{\rm{s}}\,{\rm{a}}{\rm{n}}{\rm{d}}\,\text{MTL})\end{array}.$$

For completeness, the boundary equations between the lumped parameter circuit and the MTL would be expressed as

44 $$\begin{array}{c}\{(\begin{array}{cc}-{{\bf{D}}}_{{\rm{V}}{\rm{C}}{\rm{V}}{\rm{S}}} & -{{\bf{D}}}_{{\rm{C}}{\rm{C}}{\rm{V}}{\rm{S}}}\\ {{\bf{D}}}_{{\rm{V}}{\rm{C}}{\rm{C}}{\rm{S}}} & {{\bf{D}}}_{{\rm{C}}{\rm{C}}{\rm{C}}{\rm{S}}}\end{array})+(\begin{array}{cc}{{\bf{A}}}^{T} & -{\bf{Z}}\\ {\bf{0}} & {\bf{A}}\end{array})\}(\begin{array}{c}{{\bf{U}}}^{m+1}\\ {{\bf{I}}}^{m+1}\end{array})\\ \,=\{(\begin{array}{cc}{{\bf{D}}}_{{\rm{V}}{\rm{C}}{\rm{V}}{\rm{S}}} & {{\bf{D}}}_{{\rm{C}}{\rm{C}}{\rm{V}}{\rm{S}}}\\ {\bf{0}} & {\bf{0}}\end{array})-(\begin{array}{cc}\varepsilon {{\bf{A}}}^{T} & -{\boldsymbol{\delta }}{\bf{Z}}\\ {\bf{0}} & {\bf{0}}\end{array})\}(\begin{array}{c}{{\bf{Z}}}^{m}\\ {{\bf{I}}}^{m}\end{array})+(\begin{array}{c}{{\bf{E}}}^{m+1}+{{\bf{E}}}^{m}\\ -{{\bf{A}}}_{J}{{\bf{J}}}^{m+1}\end{array}).\end{array}$$

Here, the potential **U**^*m* + 1^ and the current **I**^*m* + 1^ contain both those of the lumped parameter circuit and the MTL as written in Eqs (36) and (37). The time dependent IPE (44) expresses the boundary conditions of the lumped parameter circuit and the MTL equations, which include terms treating radiation.

We are now able to perform numerical calculations for various problems. In particular, it is important to analyze three line circuit for the discussion of the electromagnetic noise. In the three-line system, there are three modes expressed by (*U*_*i*_, *I*_*i*_) for *i* = 1, 2, 3. Hence, there are three modes to be expressed using the normal mode, and common mode, and additionally the antenna mode^15^. We should first calculate the potentials and currents in the present formalism, and take linear combinations to get the normal, common and antenna modes:

45 $$\begin{array}{c}\begin{array}{rcl}{U}_{n} & = & {U}_{1}-{U}_{2},\\ {U}_{c} & = & \frac{1}{2}({U}_{1}+{U}_{2})-{U}_{3},\\ {U}_{a} & = & \frac{1}{2}(\frac{1}{2}({U}_{1}+{U}_{2})+{U}_{3}),\\ {I}_{n} & = & \frac{1}{2}({I}_{1}-{I}_{2}),\\ {I}_{c} & = & \frac{1}{2}({I}_{1}+{I}_{2}-{I}_{3}),\\ {I}_{a} & = & {I}_{1}+{I}_{2}+{I}_{3}.\end{array}\end{array}$$

Here, the potentials and currents with the suffix *n* denote those of the normal mode, suffix *c* the common mode, and suffix *a* the antenna mode^15,16^. We are able to calculate the amount of the common mode and its coupling to the normal mode. We should reduce the effects of the common and antenna modes on the normal mode to reduce the noise.

## Conclusion

We have derived equations for the treatment of any lumped parameter circuits and MTL systems for the analysis of electromagnetic noise in the time domain. We have developed the boundary conditions between lumped parameter circuits and MTL systems by introducing the time-domain IPE. The incidence matrix **A** express the Kirchhoff’s current law for a MTL and lumped parameter circuit system, and the time-domain impedance matrix **Z** for the electric elements. We do not write explicitly the nonlinear devices as diodes and transistors^24^, but we use the same method as the SPICE. As an important ingredient we explicitly write the dependent voltage and current sources in the IPE. It should be noted that our approach does not require the concepts of I-Graph or V-Graph matrices^23^.

Since our method is based on Maxwell’s equations for the MTL equations and Kirchhoff’s current and voltage laws (KCL and KVL), and the branch constitutive equation (BCE), we believe that the discretized Eqs (30) and (31), and the boundary condition equations in terms of the time-dependent IPE (44) can be used to evaluate the electromagnetic noise for antenna processes and cross talks in various complex situations. In this formalism it is straightforward to obtain the electromagnetic fields from the scalar and vector potentials around the MTL system. With this algorithm, we have already shown the relation between the common and normal modes and the origin of the heat in the circuit due to the common–normal mode conversion^25^. In addition, we have proposed a noiseless power line^26^. We can extend the present formalism to two- or three-dimension cases.
